# Myositis interstitial lung disease and autoantibodies

**DOI:** 10.3389/fmed.2023.1117071

**Published:** 2023-06-13

**Authors:** Shire Chaudhry, Lisa Christopher-Stine

**Affiliations:** ^1^Department of Medicine, Luminis Health Anne Arundel Medical Center, Annapolis, MD, United States; ^2^Division of Rheumatology, Johns Hopkins University School of Medicine, Baltimore, MD, United States

**Keywords:** myositis, interstitial lung disease, myositis specific autoantibodies, pulmonary fibrosis, narrative review

## Abstract

The aim of this review is to examine and evaluate published literature associated with idiopathic inflammatory myopathies (IIM) and interstitial lung disease (ILD) based on myositis specific autoantibodies (MSA) and the potential clinical significance of each autoantibody subtype for the practicing clinician. The review is a comprehensive search of literature published in PubMed from the year 2005 and onward coinciding with the surge in the discovery of new MSAs. Additionally, we comment on recommended multidisciplinary longitudinal care practices for patients with IIM-ILD with regard to imaging and other testing. Treatment is not covered in this review.

## Introduction

Idiopathic inflammatory myopathies (IIM) are a diverse group of autoimmune inflammatory conditions with multi-organ system involvement. Historically, our understanding of IIM was limited to two broad classifications, dermatomyositis (DM) and polymyositis (PM). The spectrum of IIM has further evolved since the discovery of myositis specific auto-antibodies, yielding new subsets of IIM with distinct clinical, histopathological, and radiologic features aiding in our understanding of the various clinical phenotypes of disease and helping prognosticate organ involvement. Currently, IIM is broadly delineated into dermatomyositis, immune mediated necrotizing myopathy, inclusion body myositis, and overlap syndrome which are further subcategorized on the basis of individual myositis specific autoantibodies. Although they are termed myopathies (often interchangeable with the term “myositis”), they present with varying clinical manifestations. Extra-muscular involvement, including the lungs, skin, joints, and the gastrointestinal tract are among a few organs involved, exemplifying the systemic nature of the disease. Lung involvement can be catastrophic and may lead to mortality. Interstitial Lung Disease (ILD) has been associated with IIM, but it is also recognized that while associated with myositis-specific autoantibodies ILD may be the predominant or sole phenotypic element of the syndrome, with little to no myopathic symptoms present.

Idiopathic inflammatory myopathies are rare diseases that have an extensive range of estimates in determining the incidence and prevalence of IIM whether within the United States and/or worldwide. The determination of incidence and prevalence is multifactorial and may depend on the presentation of patients to specialty centers for accurate diagnosis and continuous monitoring as well as accurate reporting of these diagnoses *via* diagnostic International Classification of Diseases (ICD) codes, which may pose as a challenge with the discovery of new subgroups of IIM and new clinical classifications. As a comparison, Furst et al. determined the adjusted annual incidence of IIM to be 5.8–7.9 per 100,000 person-years, and prevalence ranged from 14 to 17.4 per 100,000 in the United States from the years 2003–2008. Furst et al. (1) A Swedish study surveying its national registrar estimated the incidence of IIM to be 11 per 1,000,000 person years and prevalence of IIM to be 14 per 100,000. Svensson et al. (2) A Korean population study estimated the incidence of IIM to be 2.9–5.2 per 1,000,000 person-years and prevalence of IIM to be 2.3–4 per 100,000. Cho et al. (3) The above data exemplifies the vast incidence and prevalence rates of IIM and while we were unable to find recently evaluated rates for global incidence and prevalence of IIM, we suspect that national rates may be underestimations of the true incidence and prevalence of disease given the challenging nature of disease presentation, especially when IIM may manifest with extramuscular manifestations of the disease. The global incidence and prevalence of interstitial lung diseases was recently investigated by Kaul et al. in 2022 and they found that the estimated global incidence of ILD ranged from 1 to 31.5 per 100,000 person-years and prevalence ranged from 6.3 to 71 per 100,000 people (4) whereas the global prevalence of ILD-IIM has risen significantly from an estimated 5% in 1974 (5) to 41% (6) of ILD cases as reported in a meta-analysis examining patients over a course of 20 years (7).

IIMs, particularly dermatomyositis, immune mediated necrotizing myopathy, and overlap syndromes are more prevalent in women whereas inclusion body myositis is seen more commonly in men (2, 8–11). A United States cohort assessment found an increasing incidence of IIM occurring in the fifth and sixth decades of life (1).

The clinical manifestations of IIM are heterogeneous and can present with acute, subacute, or chronic symptoms. Typically, myopathic symptoms such as complaints of symmetrical proximal muscle weakness usually evoke clinicians to suspect IIM, however, the initial presentations of IIM can be clinically amyopathic. Accompanying signs and symptoms may vary between the inflammatory myopathy subtypes, such as the presence of dermatological signs and symptoms in dermatomyositis which may include a heliotrope rash, gottron papules, V-sign rash, lateral thigh or holster rash, mechanic’s hands, alopecia, and calcinosis; presence of dysphagia in immune mediated necrotizing myopathy and inclusion body myositis, or presence of subtle findings such as atrophy of wrist and finger flexors in inclusion body myositis (12).

The extramuscular manifestations and multi-system organ involvement are particularly important for a practicing clinician to remain cognizant of, as IIM can involve the cardiovascular system, gastrointestinal system, skeletal system, and pulmonary system of which interstitial lung disease is a common manifestation. The signs and symptoms of ILD may present with cough, dyspnea, exercise intolerance, digital clubbing, and signs of pulmonary hypertension such as an accentuated closure of the pulmonic valve on cardiac valve auscultation (13). In IIM-ILD, pulmonary involvement may present in tandem to or subsequently after the myocutaneous manifestations of disease, however, it is of importance to note that pulmonary involvement may be the leading presentation of disease without concomitant myopathic or cutaneous manifestations, therefore, necessitating a high index of suspicion for underlying rheumatologic processes when evaluating ILD as a sole presenting manifestation (14, 15).

Diagnostic evaluation of IIM-ILD entails clinical suspicion from patient history and physical examination in combination with serologic testing (of which negative serologies may not exclude disease), radiologic testing, invasive testing through tissue biopsy, and multidisciplinary evaluations to rule out other conditions. We discuss and comment on individual diagnostic evaluations of IIM-ILD by individual myositis specific auto-antibodies in this review.

Management of IIM-ILD is particularly challenging for patients and clinicians due to the varying clinical presentations of disease and the potential of multi-systemic organ involvement coupled with a paucity of standard treatment regimens which generates variable treatment practices among providers. Additionally, most treatment guidance is from retrospective cohort studies, while only a few randomized controlled studies exist (16). The initial treatment approach usually begins with glucocorticoids, which may or may not help patients in attaining functional improvement, and the chronic use of glucocorticoids is also limited due to its adverse effects and long term complications. The data supporting glucocorticoid use is variable and is mostly based on historical precedent with scant prospective evidence supporting its use (16–18). Subsequent therapy options include immunosuppressive therapy and biologic agents, salvage therapy, and even intravenous immunoglobulins and plasma exchange, which all have varying levels of clinical evidence and benefit which can vary with specific myositis specific autoantibodies (14).

Overall, the development of ILD in patients with IIM portends a poorer prognosis with an increased risk for mortality; clinical outcomes are variable in part due to the unknown response to treatment in individual patients and in part to varying prognosis among individual myositis specific autoantibodies (16). The presence of certain autoantibodies such as anti-MDA5, anti-Jo-1, and anti-Ro-52 have been associated with an increased risk of mortality. Interestingly, while malignancy has been associated with a poorer prognosis in myositis, patients who develop malignancy are at decreased risk of developing ILD (14, 16).

## Methods

**Figure 1:**
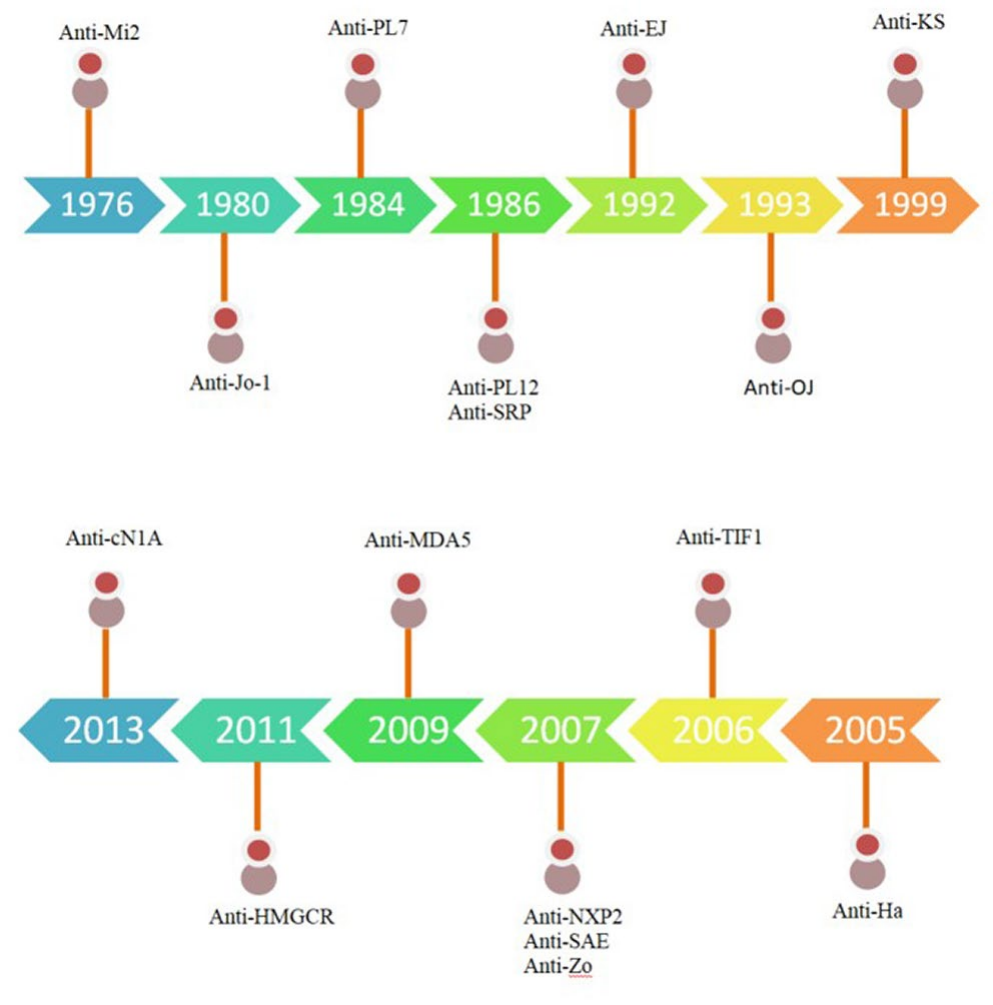
Timeline of Myositis specific auto-antibodies. Adapted from Neil J. McHugh and Sarah L. Tansley.

This comprehensive review aims to analyze and review literature by searching the electronic medical database Pubmed for the following keywords that were chosen due to their established association with our topic of interest: (12, 15, 19, 20) *idiopathic inflammatory myopathy, dermatomyositis (and individual MSA’s: anti-MDA5, anti-NXP2, anti-Mi-2, anti-TIF1γ, anti-SAE), immune mediated necrotizing myopathy (and individual MSA’s: anti-HMGCR, anti-SRP), inclusion body myositis, and overlap syndrome (and individual MSA’s: anti PM/Scl, anti-Ku, anti-RNP and anti-Ro, anti-synthetase antibodies and its entities)* each in combination with *interstitial lung disease, nonspecific interstitial pneumonia, usual interstitial pneumonia, and organizing pneumonia,* from the year 2005 and onward (Figure 1).

## Idiopathic inflammatory myopathy classification

First described in the literature by Wagner and Unverricht as early as 1863, dermatomyositis (DM) and polymyositis (PM) criteria were not established until 1975 by Bohan and Peter (21–24). Their criteria focused on the presence or absence of clinical manifestations of muscle weakness, elevation of serum markers of skeletal muscle enzymes, characteristic findings of myopathy on electromyography, select muscle biopsy findings, and the presence of typical cutaneous changes to categorize IIM into DM or PM based “definite,” “probable,” or “possible” diagnoses (23, 24). Over the next few decades, the discovery of myositis specific autoantibodies led to remarkable progress in the understanding of the pathophysiologic processes behind IIM and allowed for the creation of entities and subsets within IIM that better represented individual disease manifestations. Thus, the discovery of myositis specific auto-antibodies ultimately necessitated a reconstruction of the current framework on the approach to IIM and led to the 2017 European League Against Rheumatism (EULAR) and American College of Rheumatology (ACR) classification criteria (25). For the scope of this review, we categorize IIM into the following categories: Dermatomyositis (DM), Immune Mediated Necrotizing Myopathy (IMNM), Antisynthetase syndrome (ASynS), and Overlap Myositis (OM). The term “polymyositis” has fallen out of favor recently, with the recognition that this term was often comprised of those with overlap myositis, inclusion body myositis, the antisynthetase syndrome without a rash, or a mimic of myositis, such as muscular dystrophy (26, 27). Thus, polymyositis is not included here. Additionally, inclusion body myositis does not have associated interstitial lung disease and is therefore also not included (14). It must be noted that neither Bohan and Peter nor the ACR/EULAR criteria include pulmonary symptoms as part of the formal classification criteria.

## Interstitial lung disease

The reported global prevalence of ILD is likely as high as 41% in patients diagnosed with IIM based on a meta-analysis analyzing 34 studies with a cohort of 10,130 patients over a 20-year period (6). High resolution CT scan (HRCT) is a non-invasive diagnostic modality that is often used to assess pulmonary involvement and is considered the gold standard. Idiopathic interstitial pneumonia can be further classified as idiopathic pulmonary fibrosis which presents with a usual interstitial pneumonia (UIP) pattern or as nonspecific interstitial pneumonia (NSIP) which may mimic interstitial pulmonary fibrosis (28). A diagnosis of UIP can be made with the presence of subpleural or basal honeycombing and by identifying a reticular pattern of fine lines. Additionally, the presence of peripheral traction bronchiectasis represents lung fibrosis and can be used as a prognostic indicator (29). NSIP is the second most common presenting pattern of idiopathic interstitial pneumonia after UIP and can be challenging to distinguish due to features that overlap with UIP patterns, however, the absence of honeycombing, subpleural sparing, and presence of ground glass opacities (GGOs) are more consistent with NSIP (28, 29). A meta-analysis by Ebner et al., determining CT patterns and clinical features to distinguish UIP and NSIP found that in the general population, UIP patterns were more prevalent in elderly male patients with a history of smoking whereas NSIP patterns were seen more often in younger female patients who smoke less often (28). A multi-center retrospective study in 2020 sought to assess organizing pneumonia (OP) patterns based on CT scans in patients with COVID-19 and identified GGOs as the predominant manifestation on imaging followed by variations of mixed abnormalities including GGOs and consolidations with the presence or absence of linear opacities (30).

Up to 25% of patients with symptoms and signs concerning for an autoimmune disease do not meet the classification criteria for connective tissue disease (CTD) as per the American College of Rheumatology, and up to 20% of patients with idiopathic interstitial pneumonias have symptoms and clinical findings suggestive of underlying systemic processes (31). In 2015, the European Respiratory Society/American Thoracic Society developed a task force for “Undifferentiated Forms of CTD-associated ILD” and proposed a research classification of idiopathic pneumonia with autoimmune features (IPAF) to help guide further understanding of these patients (32). The current criteria to diagnose IPAF includes radiological or histopathological evidence of interstitial pneumonia and complete clinical evaluation excluding other etiologies for interstitial pneumonia, and incomplete features of a defined connective tissue disease, in addition to features from a clinical domain, serologic domain, and morphologic domain (32, 33).

**Figure 2:**
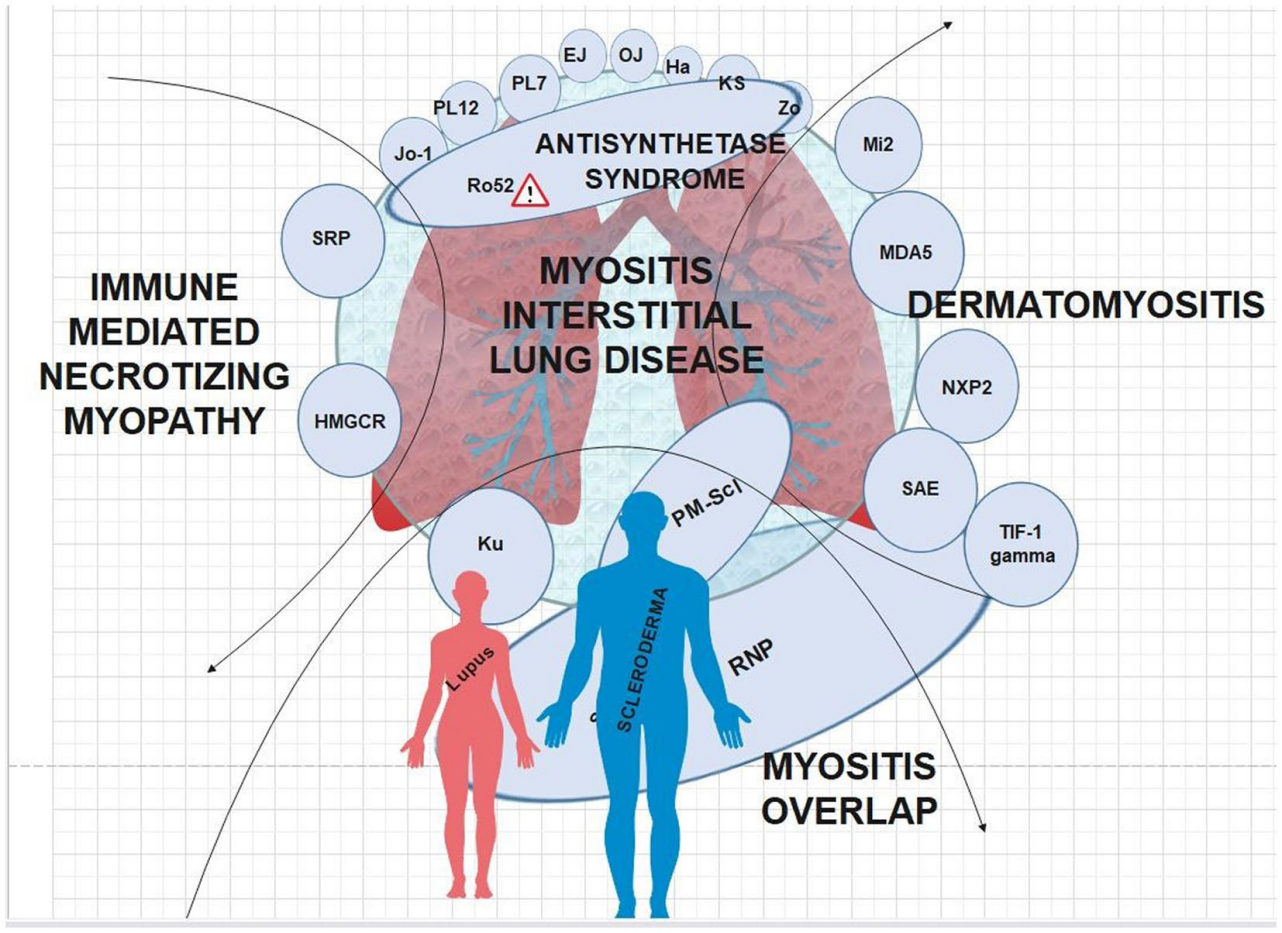
MSA’s interposed onto the IIM’s; caution symbol denotes more severe disease when anti-Ro autoantibodies are present.

There are clear clinical phenotypes with regard to certain MSA’s and IIM-ILD manifestations (Figure 2). Similar to previous reviews, our review found all anti-aminoacyl tRNA synthetase (ARS) antibody subtypes and anti-MDA5 antibodies to be associated with an increased risk of ILD relative to other MSA’s (34–36). The clinical course of anti-ARS-associated ILD appears to be generally more indolent and chronic with significantly lower rates of rapidly progressive ILD compared to anti-MDA5 (37). Previously, it has been noted that the HRCT pattern most associated with ARS antibodies was NSIP representing approximately two-thirds of cases (35). The patterns on HRCT and restrictive pattern pulmonary function tests (PFTs) are largely consistent across ARS autoantibody subtypes, and non-Jo-1 ARS autoantibodies are associated with later diagnosis, increased pulmonary fibrosis, and worsened prognosis (35).

Additional imaging modalities to screen for ILD include chest x-rays (CXR) and lung ultrasound. CXR is an easily attainable and economical imaging study that has less exposure to ionizing radiation when compared to a HRCT but has an overall decreased sensitivity in detecting ILD in comparison to the HRCT (38). Ultrasound imaging is also easily attainable and equally economical similar to CXR, moreover, it does not pose a risk to ionizing radiation exposure, and point-of-care ultrasounds can be a quick method for bedside assessment of lung parenchyma; however, it is operator dependent. Assessment of ILD through the combined use of CXR and lung ultrasound may decrease the overall exposure of patients to HRCT, therefore, Vizioli et al., compared the accuracy of combined diagnostic testing through CXR and lung ultrasound in comparison to HRCT in a single center study and concluded that lung ultrasound was highly sensitive (92%) but not specific, (79%) whereas, CXR was highly specific (91%) but not as sensitive (48%) in the detection of ILD (38). Consideration of a step-wise diagnostic approach through the use of CXR, lung ultrasound, and PFTs may be beneficial when screening for ILD in IIM.

It is our common practice to initially screen patients for ILD with HRCT and then follow their course with serial PFTs in the absence of serial imaging if they are in a high risk autoantibody group; it should be noted that serial imaging can expose patients to additional radiation and therefore should be considered when pulmonary function testing continues to show worsening of disease but not as a routine yearly screening tool. IIM-ILD is often cared for in the context of a multidisciplinary care team. If the muscle and/or skin disease is clinically significant, the patient may see only a rheumatologist, neurologist and/or dermatologist. Clinically significant interstitial lung disease requires care by a pulmonologist with specialized training. IIM-interstitial lung disease can be present in the context of dermatomyositis, the anti-synthetase syndrome, overlap myositis, or, less frequently, immune mediated necrotizing myopathy.

## Dermatomyositis

Dermatomyositis (DM) has been associated with 5 myositis-specific autoantibodies. They include anti-melanoma differentiation associated protein (anti-MDA5), antinuclear matrix protein (anti-NXP-2), anti-Mi-2, anti-transcription intermediary factor 1-𝛄 (anti-TIF1𝛄), and anti-small ubiquitin like modifier activating enzyme (anti-SAE).

### Anti-MDA5

Anti-MDA5 antibodies were first identified in Japanese patients with clinically amyopathic dermatomyositis, per Sato et al. in 2005 (39). Recent literature estimates anti-MDA5 positivity in 10–30% of all DM patients (40). Clinical manifestations of dermatomyositis are variable among certain myositis specific autoantibodies. Cutaneous manifestations can include pathognomonic findings such as Gottron’s papules, Gottron’s sign, and heliotrope rash; characteristic findings such as shawl sign, V sign, holster sign, nailfold changes, and scalp involvement; or less common and unique findings calcinosis cutis, mechanic’s hands, and panniculitis (40).

Some mucocutaneous manifestations are unique to anti-MDA5-associated DM and can present with cutaneous ulcerations in up to 82% of cases with a penchant for developing on existing gottron papules, nail folds, and overlying existing erythematous macules on extensor surfaces. Palmar papules, sometimes referred to as “inverse gottron papules,” and panniculitis are also unique findings, and if seen, should incline clinicians to suspect anti-MDA5 positivity. In addition, oral ulcers and diffuse non-scarring alopecia have an associated high prevalence. Moreover, it is thought that this peculiar constellation of findings may be due to underlying vasculopathy (41). Patients with anti-MDA5 can have muscle weakness, however, most patients have mild muscular involvement and often no muscular involvement at all, characterized as clinically amyopathic dermatomyositis (CADM) (12).

Of the 5 myositis specific autoantibodies, anti-MDA5 has been most strongly associated with ILD which portends an increased risk for mortality (Table 1). Patients with anti-MDA5 may develop features of ILD associated with classic DM or develop the life-threatening rapidly progressive subtype of ILD (RP-ILD). There is an increased prevalence of anti-MDA5 DM in Asian patients in comparison to US and European cohorts (47). The presence of anti-MDA5 is associated with the development of ILD with a reported prevalence ranging from 42 to 100% (41) with an increased predilection for development in Asian cohorts (47).

**Table 1:** Anti-MDA5 and ILD case reports.

Case no	Author year	Antibody	ILD findings	Biopsy	PFT	Age/Sex	Cohort
1.	Sato et al. (2011) (42)	MDA5	B/L lower lung interstitial changes and GGOs on CXR and HRCT RP-ILD	–	FVC: 62%	56/F	Japan *n* = 1
2.	González-Moreno et al. (2018) (43)	MDA5	Peripheral GGO at lung bases RP-ILD	Transbronchial: diffuse alveolar damage	–	54/F	Senegal *n* = 1
3.	Kaenmuang et al. (2021) (44)	MDA5 (6/6) Ro-52 (3/6) Mi-2 beta (1/6)	Subpleural involvement (5/6) GGOs (5/6) RP-ILD (4/6)	Organizing pneumonitis, focal organizing pattern, BOOP	FVC: 62, 58% DLCO: 72, 45%	Age range: 35–63 M (3/6), F (3/6)	Thailand *n* = 6
4.	De Backer et al. (2017) (45)	MDA5	Diffuse subpleural and peribronchial infiltrates and parenchymal consolidations RP-ILD	Transbronchial: diffuse alveolar damage	Restrictive with reduced DLCO	55/M	Belgium *n* = 1
5.	Li et al. (2020) (46)	MDA5 PL-7	Bilateral diffuse ground glass patchy opacities RP-ILD	–	–	27/F	Hispanic/USA *n* = 1

A review in 2020 compared the features of classic DM with ILD to anti-MDA5 DM with ILD and found that classic DM with ILD is slowly progressive, with a relapsing–remitting course, that clinically manifests with bilateral peribronchovascular ground glass opacities (GGO) or consolidations on CT scan, and nonspecific interstitial pneumonia (NSIP) or organizing pneumonia (OP) on histopathology. In comparison, RP-ILD has an epidemiologic prevalence in Asian countries, is rapidly progressive with a higher mortality rate that clinically manifests with bilateral GGO or consolidation in the posterior and peripheral lungs with the presence of diffuse alveolar damage and microangiopathy on histopathology (48). Intriguingly, the presence of anti-MDA5 antibodies is associated with an increased risk of mortality in Asian patient cohorts. Takada et al. reported a case of a 41-year-old Japanese female with clinically amyopathic dermatomyositis complicated by RP-ILD, and in an effort to increase disease awareness compared the clinical features of DM in Japanese patients with patients in the United States. The study’s findings suggest that greater than 90% of Japanese patients develop complications from ILD (of which approximately 80% of patients develop the rapidly progressive subtype), whereas only 50% of American patients with anti-MDA5 develop ILD, with fewer patients developing RP-ILD (49).

A recent multicenter retrospective cohort study of non-Asian patients from European and American centers assessed 149 patients with MDA5 DM of which 72% of patients developed ILD and only 21.5% of patients developed RP-ILD (50), comparatively, fewer patients developed RP-ILD in this cohort compared to the higher incidence of development in Asian cohorts (49, 51). Clinical manifestations also varied in this cohort with 56% of patients exhibiting muscular involvement, whereas Asian cohorts have a higher predilection for amyopathic disease (50). Consistent with our current knowledge of ILD in MDA5, the non-Asian cohort was predominantly found to have NSIP on HRCT, followed by an OP pattern. Of note13% of patients had a UIP pattern on imaging which is a less common pattern seen with MDA5; however, this reflects the diversity of disease manifestations and should remind clinicians to not anchor on the presence or absence of certain findings and to interpret objective data comprehensively (50).

The presence of certain features has been associated with the development of ILD and furthermore, poor prognosis and increased risk for mortality. There is a strong association between MDA5 and the development of cutaneous ulcerations as discussed previously. Intriguingly, the presence of cutaneous ulcers may be indicative of the presence or development of ILD. A retrospective study of 152 DM patients at Stanford University found that a majority of patients with anti-MDA5 antibodies who developed ILD also had cutaneous ulcers (52). While the presence of anti-MDA5 antibodies is associated with increased risk for the development of ILD and consequently increased risk for mortality, Chen et al. (53) the concomitant presence of anti-Ro-52 antibodies is associated with worse outcomes, increased risk of progression to RP-ILD, and decreased rates of survival as evidenced in recent Asian cohorts studies (54–56).

Anti-MDA5-associated dermatomyositis is one of the deadliest and severe ILD phenotypes in the IIMs when present. It may present with hypomyopathic or amyopathic DM. While out of the scope of this review, early and aggressive treatment is imperative; thus, early recognition is paramount, and knowledge of the unique mucocutaneous disease features may be the clinician’s first clue to diagnosing the disease.

### Anti-NXP2

Anti-NXP2 antibodies, previously reported in the literature as anti-MJ antibodies, are more commonly seen in juvenile dermatomyositis with a reported prevalence ranging from 20 to 25% in comparison to adult cohorts where the reported prevalence is 14–25% in the United States adult IIM population and 2–5% in the adult Japanese IIM population (57). Clinical features of dermatomyositis in the presence of anti-NXP2 antibodies can include the development of characteristic cutaneous manifestations, calcinosis cutis (which is prevalent in up to 37% of patients) as well as an increased prevalence of peripheral edema (58). While dermatomyositis is conventionally considered to be a disease to affect proximal muscles and cause proximal muscle weakness, anti-NXP2-associated dermatomyositis has been reported to also affect distal muscles as well causing distal arm and leg weakness. Additionally, these patients can develop symptoms of dysphagia, reflective of significant myopathic involvement (59).

In contrast to anti-MDA5 DM, pulmonary manifestations are relatively scarce, and development of ILD is rare; however, cases do exist (Table 2) (62, 63). A retrospective case series of 7 adult DM patients in France observed pulmonary involvement in 2/7 patients; PFTs of 6/7 patients observed a mean FVC of 90% and a mean DLCO of 56%. HRCT revealed NSIP in one patient, OP in one patient, and normal HRCT in four patients (60). Similarly, in a longitudinal cohort study of anti-NXP2 positive patients in the United States, only 7% of patients developed ILD with a reported mean FVC of 87%, unfortunately, this study did not discuss whether these patients underwent pulmonary imaging (59). The findings reflected in France and the United States are also similarly reflected in a Chinese cohort that identified 17 patients with anti-NXP2 antibodies of which 5 patients developed ILD in a predominantly mixed NSIP + OP pattern on HRCT (64).

**Table 2:** Anti-NXP2 and ILD.

Case no	Author year	Antibody	ILD type	ILD findings	Biopsy	PFT	Age/Sex	Cohort
1.	Bermudez et al. (2020) (60)	NXP2	NSIP: 1/6 OP: 1/6	–	–	Mean FVC: 90% ±14% Mean DLCO: 56% +/−17%	Mean age 55 +/− 13 years 5 Female 2 Male	France *n* = 7
2.	Gossez et al. (2015) (61)	NXP2	–	Bilateral consolidations lower lung zones	–	–	41 years/Female	France *n* = 1

Yan et al. performed a retrospective analysis of 33 patients with anti-NXP2 DM over a course of approximately 3 years in which 14/33 individuals developed ILD with 11/14 manifesting features of NSIP and/or OP in lung imaging (65). Interestingly, Kaplan–Meier survival curves did not reveal a statistically significant association between ILD and all-cause mortality (65). In comparison, Li et al. found 21 patients out of 70 patients to have ILD, none developing RP-ILD, in their retrospective 10 year longitudinal cohort study in China (66).

There is an association between anti-NXP2 antibodies and malignancy that was most notably reported in a Japanese cohort of adult patients in which ~37% of patients were found to have malignancy. Their findings were similar to a United States cohort study which found malignancy among ~24% of patients, however, definite associations were not exhibited (67, 68). Moreover, a recent United States cohort study from our cohort at Johns Hopkins and an DM cohort at Stanford determined that patients with anti-NXP2 antibodies are at increased risk of malignancy when compared to the general population (59).

While anti-NXP2 autoantibodies are associated with an increase in malignancy, they do not appear to have an increased association with ILD. This finding supports the observation that malignancy and ILD are inversely proportional to each other and those autoantibodies associated with a higher risk if malignancy have a lower risk of ILD.

### Anti-Mi-2

The presence of anti-Mi-2 antibodies in adults ranges from 2 to 38% among dermatomyositis (57). Patients with anti-Mi-2 antibodies predominantly present with the classic cutaneous manifestations of dermatomyositis including heliotrope rash, V sign, shawl sign, gottron papules and gottron sign, additionally, these patients can develop cuticular overgrowths (69). A recent longitudinal cohort study in our center in the United States found that the presence of anti-Mi-2 antibodies is associated with significant and persistent muscle weakness that weakly correlates with elevated creatine kinase levels (70).

Pulmonary involvement is relatively rare in anti-Mi-2 dermatomyositis with multiple cohort studies reporting minuscule lung involvement (71–73). A longitudinal study of anti-Mi-2 patients in the United States found only 3 patients out of 58 developed features of ILD (70). Literature search revealed a case report from the United States of a patient with persistent dry cough and dyspnea who was found to have bibasilar infiltrates on CXR and bilateral patchy ground glass infiltrates on HRCT, with serial imaging revealing of organizing pneumonia, in addition to the development of progressive proximal myopathy in the presence of anti-Mi-2 antibody positivity (74).

While weakness may persist in some Mi-2 + patients, overall, patients who express antibodies to anti-Mi-2 have a favorable prognosis (57, 75). Mi-2 autoantibodies are not generally associated with ILD and thus likely do not require serial PFT and other pulmonary imaging follow-up.

### Anti-transcription intermediary factor 1-𝛄

Anti-TIF1𝛄 typically manifests with more prominent cutaneous manifestations of disease and is less frequently associated with ILD. The reported prevalence of anti-TIF1𝛄 antibodies in adult dermatomyositis ranges from 13 to 31% and is more prevalent in Caucasians as compared to Asians (47, 57). Similar to other autoantibodies, cutaneous manifestations of the disease include gottron papules, heliotrope rash, and V sign, however, these patients are highly photosensitive and can present with unique cutaneous features such as ovoid palatal patches, psoriasis-like skin lesions, palmar hyperkeratosis, and hypopigmented patches overlying telangiectasias (40). In contrast, extracutaneous manifestations of the disease are less common and features of Raynaud’s phenomenon, calcinosis, arthritis/arthralgia, and pulmonary involvement are less prevalent (76).

The development of ILD is relatively uncommon with anti-TIF1𝛄 antibodies. A retrospective analysis by Harada et al. analyzed 14 patients with anti-TIF1𝛄 positivity out of a pool of 85 patients with DM over a prolonged 18-year course and identified dermatologic manifestations such as erythema, V neck sign, heliotrope rash, and nail fold telangiectasias more frequently present, whereas no patients developed features of ILD on HRCT (77). Intriguingly, patients with anti-TIF1𝛄 positivity have been found to have an increased incidence of developing malignant tumors (78, 79). Patients with anti-TIF1𝛄 and pulmonary involvement should be followed closely for the development of malignancy. Xie et al. reported a case of initial misdiagnosis of interstitial pneumonia with autoimmune features with NSIP on initial HRCT, which was identified to be right lung squamous carcinoma during a one-year follow-up (80).

Anti-TIF1𝛄 autoantibodies do not have a known association with ILD. Alternate diagnoses should be suspected if lung involvement is found in this subset of patients with DM. Again, the intriguing inverse relationship between cancer (common in this DM subset) and ILD (uncommon in TIF1𝛄 positive patients) is noteworthy.

### Anti-small ubiquitin like modifier activating enzyme

The frequency of anti-SAE antibody expression in dermatomyositis is approximately 8% (81). Patients typically present with cutaneous manifestations of the disease that precede muscle involvement (81). Extracutaneous manifestations are common, and the development of dysphagia is a frequent finding (81).

While this phenotype is more strongly associated with the dermatologic manifestations of the disease, there have been reports of mild pulmonary involvement. Gono et al., describe two case reports of Asian patients who presented with predominantly skin-related symptoms, found to have preserved pulmonary function on pulmonary function testing, but evidence of peripheral lower lobe lung involvement with subpleural ground glass opacities, more consistent with NSIP (82). In a North American cohort of 9 patients with anti-SAE positivity at the Johns Hopkins Myositis Center, 7/9 patients developed mild features of ILD, with CT findings of multiple peripheral pulmonary nodules (83). Interestingly, Kishi et al., report a case within the pediatric age group of an 8-year-old Japanese girl who presented with juvenile DM with predominantly cutaneous manifestations complicated by non-rapidly progressive ILD (84).

Overall, ILD is generally mild in these patients and improves with treatment (85). Mild ILD that seems to be clinically less significant may be a feature in patients with dermatomyositis and anti-SAE autoantibodies.

## Immune mediated necrotizing myopathy

IMNM has been associated with two prototypic autoantibodies: Anti-HMGCR (3-hydroxy-3-methylglutaryl-CoA reductase) and anti-SRP (signal recognition particle).

Anti-HMGCR antibody myopathy was identified in a US cohort of patients with necrotizing myopathy in 2010 (86) and has a reported frequency of approximately 6% (87). Patients may or may not have had exposure to statin medications and clinical manifestations can include severe proximal muscle weakness and extramuscular manifestations are mostly limited to dysphagia (87). Pulmonary involvement is uncommon with anti-HMGCR and a cohort study in China found the presence of anti-HMGCR to be a protective factor against the development of ILD (88).

Similar to anti-HMGCR antibodies, anti-signal recognition particle (SRP) antibodies are relatively rare with a reported prevalence of 4–6% in European cohorts and a slightly higher prevalence in Asian cohorts, up to approximately 13% (87). Clinical manifestations include severe proximal muscle weakness that can lead to severe debilitation, additionally, patients are at an increased risk of developing dysphagia (47).

While anti-HMGCR autoantibodies are not typically associated with interstitial lung disease, the development of ILD has been reported with anti-SRP IMNM. In a retrospective single-center study, 27 out of 60 individuals with anti-SRP IMNM were diagnosed with extra-muscular manifestations of ILD, of which the radiologic presentation of ILD was NSIP (63%), OP (33.3%), and lymphocytic interstitial pneumonia (3.7%) (89). In their cohort, opacities were primarily distributed in the lower lobes and peribronchovascular sites (89). Patients in this cohort were reported to be mostly asymptomatic with slow disease progression; they were classified as having mild to moderate severity; and none of the patients progressed to RP-ILD. Of note, patients in this cohort did not undergo confirmatory diagnostic testing with bronchoscopy or lung biopsy (89). Anti-SRP IMNM has occasionally been associated with severe forms of ILD. In a case report by Qureshi et al. a 40-year-old African American female developed ventilator-dependent respiratory failure and was found to have mildly elevated CK levels and autoantibody positivity for anti-SRP. Interestingly, the patient did not respond to corticosteroids and immunosuppressants ultimately requiring lung transplantation (90). Additionally, a literature search revealed a case report of a 29-year-old male who presented with progressive exertional dyspnea and was identified to have pulmonary arterial hypertension in addition to findings consistent with ILD. Radiographic imaging was initially consistent with an NSIP pattern with diffuse ground glass opacities but rapidly progressed to a UIP pattern with fibrotic and inflammatory changes within a mere 18 months. Ultimately, the patient underwent lung transplantation and histology from the explanted lung revealed mixed features of UIP and fibrotic NSIP (91).

IMNM is a relatively newly understood and recognized subset of IIM in the last two decades. The prototypic associated autoantibodies, anti HMGCR and anti-SRP have different predilection for ILD, with the former having no clear association and the later having a rare association but one that may be severe in nature and can present in a UIP pattern requiring lung transplantation in the most severe cases.

## Myositis overlap: anti PM/Scl, anti-Ku, anti-RNP

Myositis overlap is a heterogeneous entity in which patients can share symptoms of multiple distinct connective tissue diseases. Notable myositis overlap autoantibodies include anti-PM/Scl which generally demonstrates clinical features of overlap between scleroderma (namely Raynaud’s phenomenon, telangiectasias and possible skin thickening) and myositis with or without the skin rash of dermatomyositis.

Lung involvement is common in patients with anti-PM/Scl, ranging from 35 to 87%, and has been reported to have better functional outcomes when compared to other groups (92). In a single center study of anti-PM/Scl antibody patients in China, 30 patients were found to be positive for either anti-PM/Scl-75, anti-PM/Scl-100, or both, of which NSIP, UIP, OP, NSIP/OP overlap, and LIP were identified, respectively, in descending order of frequency through either HRCT or lung biopsy; interestingly, ILD was the sole manifesting feature in ~26% of the cohort (93).

Anti-Ku antibodies are myositis associated auto-antibodies and can be identified in patients with myositis as well as in patients with other systemic autoimmune conditions and can present with features of extramuscular involvement, such as ILD. A retrospective study seeking to identify predictive features of ILD found that within their cohort anti-ku antibodies were present in patients who developed ILD at least 12 months after the onset of their myositis, suggesting anti-ku antibodies could be associated with a slow disease progression (94). A study from the Johns Hopkins myositis cohort looking to further describe the phenotype of anti-ku positive patients found that within the cohort, ILD was the presenting feature in only 19% of patients but 56% of patients ultimately developed pulmonary disease (95).

Anti-RNP (ribonuclear protein) antibodies are prevalent in a myriad of systemic diseases such as myositis, mixed connective tissue disease, systemic lupus erythematosus, rheumatoid arthritis, and systemic sclerosis (96). A study aiming to characterize the pulmonary manifestations among patients with anti-RNP antibodies found that out of a total of 544 patients, ~25% had ILD with NSIP being the predominant radiological finding followed by UIP. Cystic lesions with ground-glass attenuation were identified in a subset of NSIP patients without signs of fibrosis on imaging, identifying an original radiologic pattern termed interstitial cystic lung disease associated with anti-RNP antibody (ICLAR) (97).

Many of the myositis CTD overlap syndromes can present with significant ILD. The practicing clinician must be aware of this potential involvement, and serial monitoring with pulmonary function testing and assessment of patient symptoms including cough and breathlessness should be closely evaluated.

## Antisynthetase syndrome

The antisynthetase syndrome is characterized by antibodies directed against an aminoacyl-transfer RNA (tRNA) synthetase (ARAs) and is associated with ILD, myositis, inflammatory arthritis, mechanic’s hands, fever, and Raynaud’s phenomenon (98). It is generally accepted that the presence of a positive antisynthetase antibody in addition to the presence of two of the following features: ILD, inflammatory myopathy, or inflammatory polyarthritis is classified as anti-synthetase syndrome (34). Alternatively, our group has proposed antisynthetase syndrome criteria that includes positive serologic testing for an anti-tRNA synthetase autoantibody in the presence of any one of the protean symptoms (ILD, myositis, inflammatory arthritis, mechanic’s hands, fever, and Raynaud’s phenomenon) (15). In a retrospective cohort of 108 patients with anti-synthetase syndrome and ILD, patients had 5 distinct antibodies, anti-Jo-1, anti-PL-7, anti-PL-12, anti-EJ, and anti-OJ (99). Thirty out of 108 cases received bronchoscopy for transbronchial biopsy to assist in pathological diagnoses, and the remaining cases were diagnosed based on radiological pattern discussions with multi-disciplinary teams (99). Data from this cohort revealed that an OP pattern was seen the most in the EJ + group, NSIP pattern was seen the most in the PL-12 + group, a mixture of OP + NSIP pattern was seen the most in the OJ + group, and UIP was seen the most in the PL-7 + group; all groups had a positive response to steroid therapy (99). In another single-center retrospective study of 84 ILD patients, the NSIP pattern was seen more in the Jo-1, PL-7, and EJ group, OP pattern was seen more in the PL-12 group, and UIP was seen more in the OJ group (100). In a retrospective cohort of 1,194 patients, patients were compared to healthy controls for the presence of anti-Ha, anti-Ks, anti-Zoα, cN1A novel myositis autoantibodies and found that the prevalence of ILD was significantly higher in those with novel myositis antibodies. Radiologic and histologic findings of UIP pattern were less frequently characterized when compared to patients with idiopathic pulmonary fibrosis (101).

Finally, anti-Ro-52 antibodies have been associated with ILD and have been considered to be an independent predictor for complications of ILD. While they may be seen in isolation, they are more often associated with other MSAs, specifically anti-ARS autoantibodies. A prospective observational study in China assessed patients with anti-Ro-52 positivity for the presence of ILD and found that patients with isolated anti-Ro-52 antibodies and non-RP-ILD had an NSIP pattern on radiographic studies whereas patients who developed RP-ILD had an OP pattern on imaging studies (102). Similarly, a retrospective analysis of ILD patients in Italy found that the presence of anti-Ro-52 antibodies could predict the development of ILD. Interestingly, patients in their cohort had statistically significant improvement in DLCO at 5 years from baseline (94).

The antisynthetase antibodies have a strong association with ILD. It must be noted that isolated ILD may be the presenting and sole feature of the illness; thus a high index of suspicion for the antisynthetase syndrome in any patient presenting with an otherwise idiopathic pneumonia, especially in an NSIP pattern, must be present. More often than not, in the antisynthetase syndrome anti-PL12 and anti-PL7 antibodies present with ILD in isolation. The unfortunate nomenclature of “myositis associated autoantibodies” can be confusing to clinicians. Thus attributing these antibodies to a syndromic complex where ILD may be the first or only symptom is an important construct to understand.

## Summary

Myositis-specific autoantibodies (MSAs) and Myositis-associated antibodies (MAAs) testing has become commercially available in recent years and is now more accessible worldwide. The diagnostic utility of the MSAs and MAAs in helping to make an accurate diagnosis and assist in the prognosis of myositis-ILD is excellent in the appropriate clinical setting. While anti-MDA5 is associated with the most severe ILD phenotype with respect to rapidly progressive ILD, many other myositis-specific and myositis-associated autoantibodies are found in conjunction with ILD in various frequencies. It is important for the practicing clinician caring for patients with myositis to recognize the significant association with ILD and appropriately triage some patients in higher-risk autoantibody-associated groups to imaging and serial pulmonary function testing for close follow-up.

## Author contributions

SC and LC-S contributed to the conception, writing, and critical review and revision of the manuscript. All authors contributed to the article and approved the submitted version.

## Funding

LC-S was supported by the Huayi and Siuling Zhang Discovery Fund.

## Conflict of interest

The authors declare that the research was conducted in the absence of any commercial or financial relationships that could be construed as a potential conflict of interest.

## Publisher’s note

All claims expressed in this article are solely those of the authors and do not necessarily represent those of their affiliated organizations, or those of the publisher, the editors and the reviewers. Any product that may be evaluated in this article, or claim that may be made by its manufacturer, is not guaranteed or endorsed by the publisher.

## References

[1] Furst DE Amato AA Iorga ŞR Gajria K Fernandes AW. Epidemiology of adult idiopathic inflammatory myopathies in a U.S. managed care plan. Muscle Nerve. (2012) 45:676–3. doi: 10.1002/mus.23302, PMID: 22499094

[2] Svensson J Arkema EV Lundberg IE Holmqvist M. Incidence and prevalence of idiopathic inflammatory myopathies in Sweden: a nationwide population-based study. Rheumatology. (2017) 56:802–11. doi: 10.1093/rheumatology/kew503, PMID: 28160487

[3] Cho SK Kim H Myung J Nam E Jung S-Y Jang EJ. Incidence and prevalence of idiopathic inflammatory myopathies in Korea: a Nationwide population-based study. J Korean Med Sci. (2019) 34:e55. doi: 10.3346/jkms.2019.34.e55, PMID: 30833879 PMC6393764

[4] Kaul B Cottin V Collard HR Valenzuela C. Variability in global prevalence of interstitial lung disease. Front Med. (2021) 8:751181. doi: 10.3389/fmed.2021.751181, PMID: 34805219 PMC8599270

[5] Frazier AR Miller RD. Interstitial pneumonitis in association with Polymyositis and Dermatomyositis. Chest. (1974) 65:403–7. doi: 10.1378/chest.65.4.403, PMID: 4819244

[6] Sun KY Fan Y Wang YX Zhong YJ Wang GF. Prevalence of interstitial lung disease in polymyositis and dermatomyositis: a meta-analysis from 2000 to 2020. Semin Arthritis Rheum. (2021) 51:175–1. doi: 10.1016/j.semarthrit.2020.11.009, PMID: 33383294

[7] Basuita M Fidler LM. Myositis antibodies and interstitial lung disease. J Appl Lab Med. (2022) 7:240–8. doi: 10.1093/jalm/jfab108, PMID: 34996093

[8] Lindgren U Pullerits R Lindberg C Oldfors A. Epidemiology survival, and clinical characteristics of inclusion body myositis. Ann Neurol. (2022) 92:201–2. doi: 10.1002/ana.26412, PMID: 35596584 PMC9541152

[9] Bernatsky S Joseph L Pineau CA Boivin DB Banerjee DB Clarke AE. Estimating the prevalence of polymyositis and dermatomyositis from administrative data: age, sex and regional differences. Ann Rheum Dis. (2009) 68:1192–6. doi: 10.1136/ard.2008.093161 18713785

[10] Betteridge Z Tansley S Shaddick G Lilleker JB Vencovsky J Chazarain L. Frequency, mutual exclusivity and clinical associations of myositis autoantibodies in a combined European cohort of idiopathic inflammatory myopathy patients. J Autoimmun. (2019) 101:48–55. doi: 10.1016/j.jaut.2019.04.001, PMID: 30992170 PMC6580360

[11] Lilleker JB Vencovsky J Wang G Wedderburn LR Diederichsen LP Schmidt J. The EuroMyositis registry: an international collaborative tool to facilitate myositis research. Ann Rheum Dis. (2018) 77:30–9. doi: 10.1136/annrheumdis-2017-211868 28855174 PMC5754739

[12] Lundberg IE Fujimoto M Vencovsky J Aggarwal R Holmqvist M Christopher-Stine L. Idiopathic inflammatory myopathies. Nat Rev Dis Primer. (2021) 7:1–22. doi: 10.1038/s41572-021-00321-x PMC10425161 34857780

[13] Antoine M Mlika M. Interstitial lung disease. StatPearls: StatPearls Publishing (2022). 31082128

[14] Lega JC Reynaud Q Belot A Fabien N Durieu I Cottin V. Idiopathic inflammatory myopathies and the lung. Eur Respir Rev. (2015) 24:216–8. doi: 10.1183/16000617.00002015, PMID: 26028634 PMC9487811

[15] Connors GR Christopher-Stine L Oddis CV Danoff SK. Interstitial lung disease associated with the idiopathic inflammatory myopathies: what progress has been made in the past 35 years? Chest. (2010) 138:1464–74. doi: 10.1378/chest.10-0180, PMID: 21138882

[16] Long K Danoff SK. Interstitial lung disease in polymyositis and dermatomyositis. Clin Chest Med. (2019) 40:561–2. doi: 10.1016/j.ccm.2019.05.004, PMID: 31376891

[17] Fujisawa T. Management of myositis-associated interstitial lung disease. Medicina (Mex). (2021) 57:347. doi: 10.3390/medicina57040347, PMID: 33916864 PMC8065549

[18] Chandra T Aggarwal R. Clinical trials and novel therapeutics in dermatomyositis. Expert Opin Emerg Drugs. (2020) 25:213–8. doi: 10.1080/14728214.2020.1787985, PMID: 32597690

[19] Satoh M Tanaka S Ceribelli A Calise SJ Chan EKL. A comprehensive overview on myositis-specific antibodies: new and old biomarkers in idiopathic inflammatory myopathy. Clin Rev Allergy Immunol. (2017) 52:1–19. doi: 10.1007/s12016-015-8510-y, PMID: 26424665 PMC5828023

[20] Halilu F Christopher-Stine L. Myositis-specific antibodies: overview and clinical utilization. Rheumatol Immunol Res. (2022) 3:1–10. doi: 10.2478/rir-2022-0001, PMID: 36467022 PMC9524809

[21] Levine TD. History of Dermatomyositis. Arch Neurol. (2003) 60:780–2. doi: 10.1001/archneur.60.5.780, PMID: 12756148

[22] Leclair V Lundberg IE. New myositis classification criteria—what we have learned since Bohan and Peter. Curr Rheumatol Rep. (2018) 20:18. doi: 10.1007/s11926-018-0726-4 29550929 PMC5857275

[23] Bohan A Peter JB. Polymyositis and Dermatomyositis. N Engl J Med. (1975a) 292:344–7. doi: 10.1056/NEJM197502132920706, PMID: 1090839

[24] Bohan A Peter JB. Polymyositis and Dermatomyositis. N Engl J Med. (1975b) 292:403–7. doi: 10.1056/NEJM197502202920807, PMID: 1089199

[25] Lundberg IE Tjärnlund A Bottai M Werth VP Pilkington C de Visser M. EULAR/ACR classification criteria for adult and juvenile idiopathic inflammatory myopathies and their major subgroups. Ann Rheum Dis. (2017) 76:1955–64. doi: 10.1136/annrheumdis-2017-211468, PMID: 29079590 PMC5736307

[26] Ramanathan RS. Polymyositis mimicking inflammatory dystrophy. ARC J Neurosci. (2017) 2:1–2. doi: 10.20431/2456-057X.0201001

[27] van der Meulen MFG Bronner IM Hoogendijk JE Voskuyl AE Dinant HJ Linssen WHJP. Polymyositis: an overdiagnosed entity. Neurology. (2003) 61:316–1. doi: 10.1212/WNL.61.3.316, PMID: 12913190

[28] Ebner L Christodoulidis S Stathopoulou T Geiser T Stalder O Limacher A. Meta-analysis of the radiological and clinical features of usual interstitial pneumonia (UIP) and nonspecific interstitial pneumonia (NSIP). PLoS One. (2020) 15:e0226084. doi: 10.1371/journal.pone.0226084, PMID: 31929532 PMC6957301

[29] Lynch DA Sverzellati N Travis WD Brown KK Colby TV Galvin JR. Diagnostic criteria for idiopathic pulmonary fibrosis: a Fleischner society white paper. Lancet Respir Med. (2018) 6:138–3. doi: 10.1016/S2213-2600(17)30433-2, PMID: 29154106

[30] Wang Y Jin C Wu CC Zhao H Liang T Liu Z. Organizing pneumonia of COVID-19: time-dependent evolution and outcome in CT findings. PLoS One. (2020) 15:e0240347. doi: 10.1371/journal.pone.0240347, PMID: 33175876 PMC7657520

[31] Karjigi U Dharmanand BG. Interstitial pneumonia with autoimmune features. Indian J Rheumatol. (2021) 16:S39. doi: 10.4103/0973-3698.332977

[32] Fernandes L Nasser M Ahmad K Cottin V. Interstitial pneumonia with autoimmune features (IPAF). Front Med. (2019) 6:209. doi: 10.3389/fmed.2019.00209 PMC6798044 31681774

[33] Karampeli M Thomas K Flouda S Chavatza A Nikolopoulos D Pieta A. Interstitial pneumonia with autoimmune features (IPAF): a single-Centre prospective study. Mediterr J Rheumatol. (2020) 31:330–6. doi: 10.31138/mjr.31.3.330, PMID: 33163866 PMC7641027

[34] Solomon J Swigris JJ Brown KK. Myositis-related interstitial lung disease and antisynthetase syndrome. Front Med. (2013) 7:16. doi: 10.3389/fmed.2020.609595 PMC3676869 21390438

[35] Gasparotto M Gatto M Saccon F Ghirardello A Iaccarino L Doria A. Pulmonary involvement in antisynthetase syndrome. Curr Opin Rheumatol. (2019) 31:603–11. doi: 10.1097/BOR.0000000000000663, PMID: 31503025

[36] Teel A Lu J Park J Singh N Basharat P. The role of myositis-specific autoantibodies and the Management of Interstitial Lung Disease in idiopathic inflammatory myopathies: a systematic review. Semin Arthritis Rheum. (2022) 57:152088. doi: 10.1016/j.semarthrit.2022.152088, PMID: 36116345

[37] Sato S Murakami A Kuwajima A Mishima M Suda T Seishima M. Clinical utility of an enzyme-linked Immunosorbent assay for detecting anti-melanoma differentiation-associated gene 5 autoantibodies. PLoS One. (2016) 11:e0154285. doi: 10.1371/journal.pone.0154285, PMID: 27115353 PMC4846082

[38] Vizioli L Ciccarese F Forti P Chiesa AM Giovagnoli M Mughetti M. Integrated use of lung ultrasound and chest X-ray in the detection of interstitial lung disease. Respiration. (2017) 93:15–22. doi: 10.1159/000452225 27880957

[39] Sato S Hirakata M Kuwana M Suwa A Shinichi M Tsuneyo M. Autoantibodies to a 140-kd polypeptide, CADM-140, in Japanese patients with clinically amyopathic dermatomyositis. Arthritis Rheum. (2005) 52:1571–6. doi: 10.1002/art.21023, PMID: 15880816

[40] DeWane ME Waldman R Lu J. Dermatomyositis: clinical features and pathogenesis. J Am Acad Dermatol. (2020) 82:267–1. doi: 10.1016/j.jaad.2019.06.1309 31279808

[41] Kurtzman DJB Vleugels RA. Anti-melanoma differentiation–associated gene 5 (MDA5) dermatomyositis: a concise review with an emphasis on distinctive clinical features. J Am Acad Dermatol. (2018) 78:776–5. doi: 10.1016/j.jaad.2017.12.010 29229575

[42] Sato S Kuwana M Fujita T Suzuki Y. Amyopathic dermatomyositis developing rapidly progressive interstitial lung disease with elevation of anti-CADM-140/MDA5 autoantibodies. Mod Rheumatol. (2012) 22:625–9. doi: 10.3109/s10165-011-0558-9 22124544

[43] González-Moreno J Raya-Cruz M Losada-Lopez I Cacheda AP Oliver C Colom B. Rapidly progressive interstitial lung disease due to anti-MDA5 antibodies without skin involvement: a case report and literature review. Rheumatol Int. (2018) 38:1293–6. doi: 10.1007/s00296-018-3991-7, PMID: 29417209 PMC7101732

[44] Kaenmuang P Navasakulpong A. Clinical characteristics of anti-MDA5 antibody-positive interstitial lung disease. Respirol Case Rep. (2020) 9:e00701. doi: 10.1002/rcr2.701 33343905 PMC7734424

[45] De Backer E Gremonprez F Brusselle G Depuydt P Van Dorpe J Van Haverbeke C. Anti-MDA5 positive dermatomyositis complicated with rapidly progressive interstitial lung disease – a case report. Acta Clin Belg. (2018) 73:413–7. doi: 10.1080/17843286.2017.1420521, PMID: 29287518

[46] Li ZY Gill E Mo F Reyes C. Double anti-PL-7 and anti-MDA-5 positive Amyopathic Dermatomyositis with rapidly progressive interstitial lung disease in a Hispanic patient. BMC Pulm Med. (2020) 20:220. doi: 10.1186/s12890-020-01256-x 32799827 PMC7429127

[47] Betteridge Z McHugh N. Myositis-specific autoantibodies: an important tool to support diagnosis of myositis. J Intern Med. (2016) 280:8–23. doi: 10.1111/joim.12451, PMID: 26602539

[48] De Lorenzis E Natalello G Gigante L Verardi L Bosello SL Gremese E. What can we learn from rapidly progressive interstitial lung disease related to anti-MDA5 dermatomyositis in the management of COVID-19? Autoimmun Rev. (2020) 19:102666. doi: 10.1016/j.autrev.2020.102666, PMID: 32942036 PMC7489246

[49] Takada T Asakawa K Barrios R. A Japanese-American female with rapidly progressive interstitial lung disease associated with clinically amyopathic dermatomyositis. Clin Rheumatol. (2021) 40:1159–65. doi: 10.1007/s10067-020-05292-0, PMID: 32676922

[50] Cavagna L Meloni F Meyer A Sambataro G Belliato M De Langhe E. Clinical spectrum time course in non-Asian patients positive for anti-MDA5 antibodies. Clin Exp Rheumatol. (2022) 40:274–3. doi: 10.55563/clinexprheumatol/di1083, PMID: 35200123

[51] Moghadam-Kia S Oddis CV Sato S Kuwana M Aggarwal R. Antimelanoma differentiation-associated gene 5 antibody: expanding the clinical Spectrum in north American patients with Dermatomyositis. J Rheumatol. (2017) 44:319–5. doi: 10.3899/jrheum.160682, PMID: 28089977

[52] Narang NS Casciola-Rosen L Li S Chung L Fiorentino DF. Cutaneous ulceration in dermatomyositis: association with anti-melanoma differentiation-associated gene 5 antibodies and interstitial lung disease. Arthritis Care Res. (2015) 67:667–2. doi: 10.1002/acr.22498, PMID: 25331610 PMC4404195

[53] Chen Z Cao M Plana MN Liang J Cai H Kuwana M. Utility of anti-melanoma differentiation-associated gene 5 antibody measurement in identifying patients with dermatomyositis and a high risk for developing rapidly progressive interstitial lung disease: a review of the literature and a meta-analysis. Arthritis Care Res. (2013) 65:1316–24. doi: 10.1002/acr.21985 23908005

[54] Xu A Ye Y Fu Q Lian X Chen S Guo Q. Prognostic values of anti-Ro52 antibodies in anti-MDA5-positive clinically amyopathic dermatomyositis associated with interstitial lung disease. Rheumatol Oxf Engl. (2021) 60:3343–51. doi: 10.1093/rheumatology/keaa786, PMID: 33331866

[55] Gui X Shenyun S Ding H Wang R Tong J Yu M. Anti-Ro52 antibodies are associated with the prognosis of adult idiopathic inflammatory myopathy-associated interstitial lung disease. Rheumatol Oxf Engl. (2022) 61:4570–8. doi: 10.1093/rheumatology/keac090, PMID: 35148366

[56] Lv C You H Xu L Wang L Yuan F Li J. Coexistence of anti-Ro52 antibodies in anti-MDA5 antibody-positive Dermatomyositis is highly associated with rapidly progressive interstitial lung disease and mortality risk. J Rheumatol. (2023) 50:219–6. doi: 10.3899/jrheum.220139, PMID: 35705235

[57] Wolstencroft PW Fiorentino DF. Dermatomyositis clinical and pathological phenotypes associated with myositis-specific autoantibodies. Curr Rheumatol Rep. (2018) 20:28. doi: 10.1007/s11926-018-0733-5 29637414

[58] Rogers A Chung L Li S Casciola-Rosen L Fiorentino DF. Cutaneous and systemic findings associated with nuclear matrix protein 2 antibodies in adult Dermatomyositis patients. Arthritis Care Res. (2017) 69:1909–14. doi: 10.1002/acr.23210, PMID: 28129490 PMC5529261

[59] Albayda J Pinal-Fernandez I Huang W Parks C Paik J Casciola-Rosen L. Antinuclear matrix protein 2 autoantibodies and edema, muscle disease, and malignancy risk in Dermatomyositis patients. Arthritis Care Res. (2017) 69:1771–6. doi: 10.1002/acr.23188, PMID: 28085235 PMC5509530

[60] Bermudez J Heim X Bertin D. Lung involvement associated with anti-NXP2 autoantibodies in inflammatory myopathies: a French monocenter series. Expert Rev Respir Med. (2020) 14:845–11. doi: 10.1080/17476348.2020.1767598, PMID: 32394768

[61] Gossez M Levesque M Khouatra C Cottin V Garnier L Fabien N. Interstitial lung disease in an adult patient with dermatomyositis and anti-NXP2 autoantibody. Eur Respir Rev. (2015) 24:370–2. doi: 10.1183/16000617.00006714, PMID: 26028648 PMC9487820

[62] Li L Wang H Wang Q Wu C Liu C Zhang Y. Myositis-specific autoantibodies in dermatomyositis/polymyositis with interstitial lung disease. J Neurol Sci. (2019) 397:123–8. doi: 10.1016/j.jns.2018.12.040 30616054

[63] Ceribelli A Fredi M Taraborelli M Cavazzana I Franceschini F Quinzanini M. Anti-MJ/NXP-2 autoantibody specificity in a cohort of adult Italian patients with polymyositis/dermatomyositis. Arthritis Res Ther. (2012) 14:R97. doi: 10.1186/ar3822, PMID: 22546500 PMC3446471

[64] Yan TT Zhang X Yang HH Sun W-J Liu L Du Y. Association of anti-NXP2 antibody with clinical characteristics and outcomes in adult dermatomyositis: results from clinical applications based on a myositis-specific antibody. Clin Rheumatol. (2021) 40:3695–02. doi: 10.1007/s10067-021-05667-x, PMID: 33712891

[65] Yan T Du Y Sun W Chen X Wu Q Ye Q. Interstitial lung disease in adult patients with anti-NXP2 antibody positivity: a multicentre 18-month follow-up study. Clin Exp Rheumatol. Published online. (2023) 41:247–253. doi: 10.55563/clinexprheumatol/lqjx4h, PMID: 35819809

[66] Li S Sun C Zhang L Han J Yang H Gao S. Clinical heterogeneity of patients with antinuclear matrix protein 2 antibody–positive myositis: a retrospective cohort study in China. J Rheumatol. (2022) 49:922–8. doi: 10.3899/jrheum.211234, PMID: 35705242

[67] Ichimura Y Matsushita T Hamaguchi Y Kaji K Hasegawa M Tanino Y. Anti-NXP2 autoantibodies in adult patients with idiopathic inflammatory myopathies: possible association with malignancy. Ann Rheum Dis. (2012) 71:710–3. doi: 10.1136/annrheumdis-2011-200697, PMID: 22258483

[68] Fiorentino DF Chung LS Christopher-Stine L Zaba L Li S Mamme AL. Most patients with Cancer-associated Dermatomyositis have antibodies to nuclear matrix protein NXP-2 or transcription intermediary factor 1γ. Arthritis Rheum. (2013) 65:2954–62. doi: 10.1002/art.38093, PMID: 24037894 PMC4073292

[69] Marvi U Chung L Fiorentino DF. Clinical presentation and evaluation of Dermatomyositis. Indian J Dermatol. (2012) 57:375–1. doi: 10.4103/0019-5154.100486, PMID: 23112358 PMC3482801

[70] Pinal-Fernandez I Mecoli CA Casal-Dominguez M Pak K Hosono Y Huapaya J. More prominent muscle involvement in patients with dermatomyositis with anti-Mi2 autoantibodies. Neurology. (2019) 93:e1768–77. doi: 10.1212/WNL.0000000000008443, PMID: 31594859 PMC6946486

[71] Gómez GN Pérez N Braillard Poccard A Gómez RA Costi AC Mercedes A. Myositis-specific antibodies and clinical characteristics in patients with autoimmune inflammatory myopathies: reported by the argentine registry of inflammatory myopathies of the argentine Society of Rheumatology. Clin Rheumatol. (2021) 40:4473–83. doi: 10.1007/s10067-021-05797-2, PMID: 34159491

[72] dos Passos Carvalho MIC Shinjo SK. Frequency and clinical relevance of anti-Mi-2 autoantibody in adult Brazilian patients with dermatomyositis. Adv Rheumatol. (2019) 59:27. doi: 10.1186/s42358-019-0071-y 31266544

[73] Srivastava P Dwivedi S Misra R. Myositis-specific and myositis-associated autoantibodies in Indian patients with inflammatory myositis. Rheumatol Int. (2016) 36:935–3. doi: 10.1007/s00296-016-3494-3, PMID: 27193471

[74] Ahmad A Attoti Y Bernstein KA. A man with recurrent pneumonitis: a rare case of interstitial lung disease associated with anti-Mi-2 Beta-specific Dermatomyositis. Cureus. (2021) 13:e20334. doi: 10.7759/cureus.20334, PMID: 35028227 PMC8747975

[75] Komura K Fujimoto M Matsushita T Kaji K Kondo M Hirano T. Prevalence and clinical characteristics of anti-Mi-2 antibodies in Japanese patients with dermatomyositis. J Dermatol Sci. (2005) 40:215–7. doi: 10.1016/j.jdermsci.2005.09.004, PMID: 16289693

[76] Fiorentino DF Kuo K Chung L Zaba L Li S Casciola-Rosen L. Distinctive cutaneous and systemic features associated with antitranscriptional intermediary factor-1γ antibodies in adults with dermatomyositis. J Am Acad Dermatol. (2015) 72:449–5. doi: 10.1016/j.jaad.2014.12.009 25595720 PMC4351728

[77] Harada Y Tominaga M Iitoh E Kaieda S Koga T Fujimoto K. Clinical characteristics of anti-TIF-1γ antibody-positive Dermatomyositis associated with malignancy. J Clin Med. (2022) 11:1925. doi: 10.3390/jcm11071925, PMID: 35407533 PMC8999723

[78] Varedi D Frigerio A Scaife C Hull C. A novel case of TIF1 gamma autoantibody positive dermatomyositis associated with a non-functional pancreatic neuroendocrine tumor. Dermatol Online J. (2019) 25:13030/qt4fc9p1bd. doi: 10.5070/D3253043339 30982303

[79] Czerwinska P Wlodarczyk NA Jaworska AM Mackiewicz AA. The association between TIF1 family members and Cancer Stemness in solid tumors. Cancers. (2021) 13:1528. doi: 10.3390/cancers13071528, PMID: 33810347 PMC8061774

[80] Xie J Jiao LB Xu R Zhi DX Zhi HJ. Anti-TIF1 gamma-positive IPAF patient developed stage IVB lung squamous carcinoma in 1 year: a case report. BMC Pulm Med. (2021) 21:204. doi: 10.1186/s12890-021-01570-y 34193090 PMC8242283

[81] Betteridge ZE Gunawardena H Chinoy H North J Ollier WER Cooper RG. Clinical and human leucocyte antigen class II haplotype associations of autoantibodies to small ubiquitin-like modifier enzyme, a dermatomyositis-specific autoantigen target, in UK Caucasian adult-onset myositis. Ann Rheum Dis. (2009) 68:1621–5. doi: 10.1136/ard.2008.097162 18930994

[82] Gono T Tanino Y Nishikawa A Kawamata T Hirai K Okazaki Y. Two cases with autoantibodies to small ubiquitin-like modifier activating enzyme: a potential unique subset of dermatomyositis-associated interstitial lung disease. Int J Rheum Dis. (2019) 22:1582–6. doi: 10.1111/1756-185X.13593, PMID: 31050194

[83] Albayda J Mecoli C Casciola-Rosen L Danoff SK Lin CT Hines D. A north American cohort of anti-SAE Dermatomyositis: clinical phenotype, testing, and review of cases. ACR Open Rheumatol. (2021) 3:287–4. doi: 10.1002/acr2.11247, PMID: 33774928 PMC8126760

[84] Kishi T Tani Y Okiyama N Mizuochi K Ichimura Y Harigai M. Anti-SAE autoantibody-positive Japanese patient with juvenile dermatomyositis complicated with interstitial lung disease - a case report. Pediatr Rheumatol Online J. (2021) 19:34. doi: 10.1186/s12969-021-00532-2 33740993 PMC7980636

[85] Alenzi FM. Myositis specific autoantibodies: a clinical perspective. Open Access Rheumatol Res Rev. (2020) 12:9–14. doi: 10.2147/OARRR.S231195, PMID: 32021502 PMC6969688

[86] Christopher-Stine L Casciola-Rosen LA Hong G Chung T Corse AM Mammen AL. A novel autoantibody recognizing 200-kd and 100-kd proteins is associated with an immune-mediated necrotizing myopathy. Arthritis Rheum. (2010) 62:2757–66. doi: 10.1002/art.27572, PMID: 20496415 PMC3026777

[87] Anquetil C Boyer O Wesner N Benveniste O Allenbach Y. Myositis-specific autoantibodies, a cornerstone in immune-mediated necrotizing myopathy. Autoimmun Rev. (2019) 18:223–11. doi: 10.1016/j.autrev.2018.09.008, PMID: 30639649

[88] Li S Ge Y Yang H Wang T Zheng X Peng Q. The spectrum and clinical significance of myositis-specific autoantibodies in Chinese patients with idiopathic inflammatory myopathies. Clin Rheumatol. (2019) 38:2171–9. doi: 10.1007/s10067-019-04503-7, PMID: 30863950

[89] Ge Y Yang H Xiao X Liang L Lu X Wang G. Interstitial lung disease is not rare in immune-mediated necrotizing myopathy with anti-signal recognition particle antibodies. BMC Pulm Med. (2022) 22:14. doi: 10.1186/s12890-021-01802-1 35000598 PMC8744320

[90] Qureshi A Brown D Brent L. Anti-signal recognition particle antibody-associated severe interstitial lung disease requiring lung transplantation. Cureus. (2020) 12:e7962. doi: 10.7759/cureus.7962, PMID: 32523819 PMC7273426

[91] Baah S Gorgone M Lachant D. Asymptomatic necrotizing myositis in a young male with progressive interstitial lung disease. Respir Med Case Rep. (2021) 32:101374. doi: 10.1016/j.rmcr.2021.101374, PMID: 33747761 PMC7972976

[92] Kuwana M Gil-Vila A Selva-O’Callaghan A. Role of autoantibodies in the diagnosis and prognosis of interstitial lung disease in autoimmune rheumatic disorders. Ther Adv Musculoskelet Dis. (2021) 13:11032457. doi: 10.1177/1759720X211032457, PMID: 34377160 PMC8320553

[93] Ge Y Shu X He L Li C Lu X Wang G. Interstitial lung disease is a major characteristic of patients who test positive for anti-PM/Scl antibody. Front Med. (2022) 8:778211. doi: 10.3389/fmed.2021.778211, PMID: 35118087 PMC8804089

[94] Vojinovic T Cavazzana I Ceruti P Fredi M Modina D Berlendis M. Predictive features and clinical presentation of interstitial lung disease in inflammatory myositis. Clin Rev Allergy Immunol. (2021) 60:87–94. doi: 10.1007/s12016-020-08814-5, PMID: 33141387 PMC7819919

[95] Casal-Dominguez M Pinal-Fernandez I Derfoul A Graf R Michelle H Albayda J. The phenotype of myositis patients with anti-Ku autoantibodies. Semin Arthritis Rheum. (2021) 51:728–4. doi: 10.1016/j.semarthrit.2021.04.012, PMID: 34144382 PMC8384675

[96] Elhani I Khoy K Mariotte D Comby E Marcelli C Le Mauff B. The diagnostic challenge of patients with anti-U1-RNP antibodies. Rheumatol Int. (2023) 43:509–521. doi: 10.1007/s00296-022-05161-w 35896805

[97] Lhote R Grenier P Haroche J Miyara M Boussouar S Mathian A. Characterization of interstitial lung disease associated with anti-Ribonucleoprotein antibodies. JCR J Clin Rheumatol. (2020) 26:327–3. doi: 10.1097/RHU.0000000000001127, PMID: 31415476

[98] Sawal N Mukhopadhyay S Rayancha S Moore A Garcha P Kumar A. A narrative review of interstitial lung disease in anti-synthetase syndrome: a clinical approach. J Thorac Dis. (2021) 13:5556–71. doi: 10.21037/jtd-20-3328, PMID: 34659821 PMC8482343

[99] Zhan X Yan W Wang Y Li Q Shi X Gao Y. Clinical features of anti-synthetase syndrome associated interstitial lung disease: a retrospective cohort in China. BMC Pulm Med. (2021) 21:57. doi: 10.1186/s12890-021-01399-5 33579248 PMC7881640

[100] Jiang M Dong X Zheng Y. Clinical characteristics of interstitial lung diseases positive to different anti-synthetase antibodies. Medicine (Baltimore). (2021) 100:e25816. doi: 10.1097/MD.0000000000025816, PMID: 34106621 PMC8133147

[101] Moll SA Platenburg MGJP Platteel ACM Vorselaars ADM Bonàs MJ Roodenburg-Benschop C. Prevalence of novel myositis autoantibodies in a large cohort of patients with interstitial lung disease. J Clin Med. (2020) 9:2944. doi: 10.3390/jcm9092944, PMID: 32933078 PMC7563342

[102] Shao C Sun Y Huang H Zhang Z Pan R Xu K. Myositis specific antibodies are associated with isolated anti-Ro-52 associated interstitial lung disease. Rheumatology. (2022) 61:1083–91. doi: 10.1093/rheumatology/keab488, PMID: 34128956

